# A Primer on Proteomic Characterization of Intercellular Communication in a Virus Microenvironment

**DOI:** 10.1016/j.mcpro.2025.100913

**Published:** 2025-01-23

**Authors:** James C. Kostas, Colter S. Brainard, Ileana M. Cristea

**Affiliations:** Department of Molecular Biology, Princeton University, Princeton, New Jersey, USA

**Keywords:** virus microenvironment, viral infection, intercellular communication, cell-to-cell communication, extracellular vesicles, secreted proteins, secretome, secreted metabolites, cell surface, surfaceome, extracellular matrix, spatial profiling, multiplexed imaging, proteomics, microenvironment labeling

## Abstract

Intercellular communication is fundamental to multicellular life and a core determinant of outcomes during viral infection, where the common goals of virus and host for persistence and replication are generally at odds. Hosts rely on encoded innate and adaptive immune responses to detect and clear viral pathogens, while viruses can exploit or disrupt these pathways and other intercellular communication processes to enhance their spread and promote pathogenesis. While virus-induced signaling can result in systemic changes to the host, striking alterations are observed within the cellular microenvironment directly surrounding a site of infection, termed the virus microenvironment (VME). Mechanisms employed by viruses to condition their VMEs are emerging and are critical for understanding the biology and pathologies of viral infections. Recent advances in experimental approaches, including proteomic methods, have enabled study of the VME in unprecedented detail. In this review article, we provide a primer on proteomic approaches used to study how viral infections alter intercellular communication, highlighting the ways in which these approaches have been implemented and the exciting biology they have uncovered. First, we consider the different molecules secreted by an infected cell, including proteins, either soluble or contained within extracellular vesicles, and metabolites. We further discuss the modalities of interactions facilitated by alteration at the cell surface of infected cells, including immunopeptide presentation and interactions with the extracellular matrix. Finally, we review spatial profiling approaches that have allowed distinguishing how specific subpopulations of cells within a VME respond to infection and alter their protein composition, discussing valuable insights these methods have offered.

## Highlights

• Review of proteomics methods for studying a virus microenvironment.
• Intercellular communication dictates the outcome of a viral infection.
• Cellular communication *via* molecules secreted by an infected cell.
• Cell-to-cell communication *via* alterations at the surface of infected cells.
• Distinguishing cell subpopulations based on their proximity to infection.

## Graphical Abstract

**Figure:**
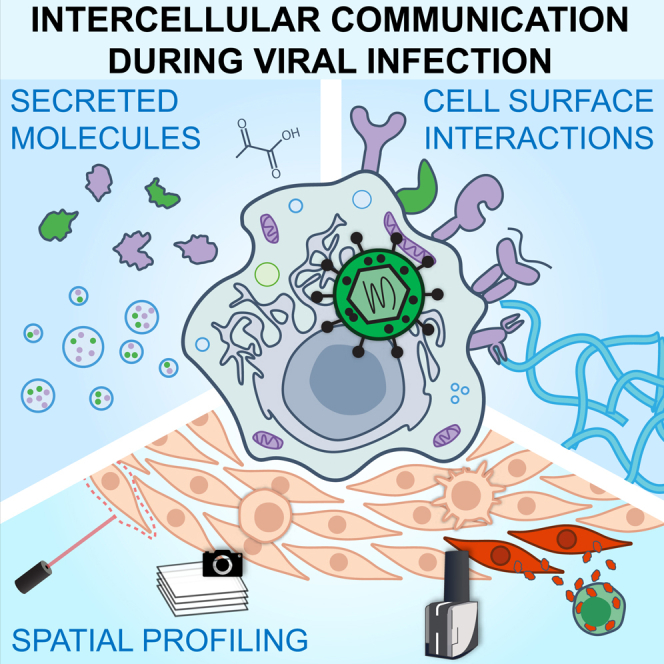

## In Brief

Intercellular communication during viral infection is at the core of virus spread and infection-induced pathologies. Cell-to-cell interactions are characterized by myriad strategies utilized by both the host and the virus, including the secretion of molecules, interactions at the cell surface, and remodeling of the extracellular matrix. In this review, we detail proteomic methods that have been developed to probe these spatial and temporal interactions, as well as highlight biological discoveries that have been made through their application.

Intercellular communication forms the basis for multicellular life. Intricate signaling mechanisms network across large numbers of cells to regulate critical biological processes, such as development, wound healing, and immunity. Cells utilize a variety of molecular cues to exchange information and coordinate responses, including soluble mediators, molecules presented at the cell surface, and extracellular matrix (ECM) components. These communication events exist in a delicate balance that, when disrupted, can cause disease. During viral infections, cell-to-cell communication is paramount. Host cells enact immune responses to clear infections, while pathogens also modulate these pathways and other intercellular communication processes to promote their survival.

From the host’s perspective, intercellular communication is essential for coordinating innate and adaptive immune responses. Infected cells and immune cells, like antigen presenting cells (APCs), detect viral components *via* pattern recognition receptors (1), triggering the release of cytokines, including interferons (IFNs). These secreted immune messenger molecules signal nearby cells to adopt antiviral states, all the while recruiting additional immune cells, like natural killer (NK) cells, to the infected tissue (2, 3, 4). NK cells can kill infected cells by recognizing missing or altered major histocompatibility complex (MHC)-I molecules on the infected cell surface (5). As an additional layer of protection, APCs present viral antigens to T cells, facilitating activation of CD4+ T helper cells, which secrete cytokines that enhance both B cell antibody production and CD8+ cytotoxic T cell activity (6). This multilayered network of signaling ensures an effective and controlled immune response that aims to clear the virus.

Viruses, through their coevolution with hosts, have also acquired mechanisms to modulate intercellular signaling. By rewiring how infected cells interface with immune cells and other cells in the surrounding tissue, viruses can reshape the cellular microenvironment in ways that promote their spread and survival within a population (7, 8, 9, 10, 11, 12, 13, 14, 15, 16, 17, 18, 19, 20, 21, 22). Specific mechanisms that are emerging are proving critical for understanding viral spread between cells and resulting pathologies. For example, the virus-driven remodeling of extracellular vesicle (EV) contents was shown to result in the delivery of viral proteins and viral RNAs to neighboring uninfected cells, priming these for infection (10, 20, 23, 24). A viral infection was also shown to direct the motility of virus-producing infected cells towards uninfected cells, as observed upon infection with vaccinia virus *via* the action of a viral epidermal growth factor homolog, promoting cell-to-cell spread of infection (21). At the interface of viral and bacterial infections, influenza A virus (IAV) infection was found to alter the airway epithelium ECM, leading to acidification of the airway surface liquid and predisposing the airway to bacterial co-infection with *Streptococcus pneumoniae* (22). These examples highlight the diversity and sophistication of the strategies acquired by viruses to modulate cellular communication and underscore the importance of studying intercellular communication during viral infection.

**Fig. 1:**
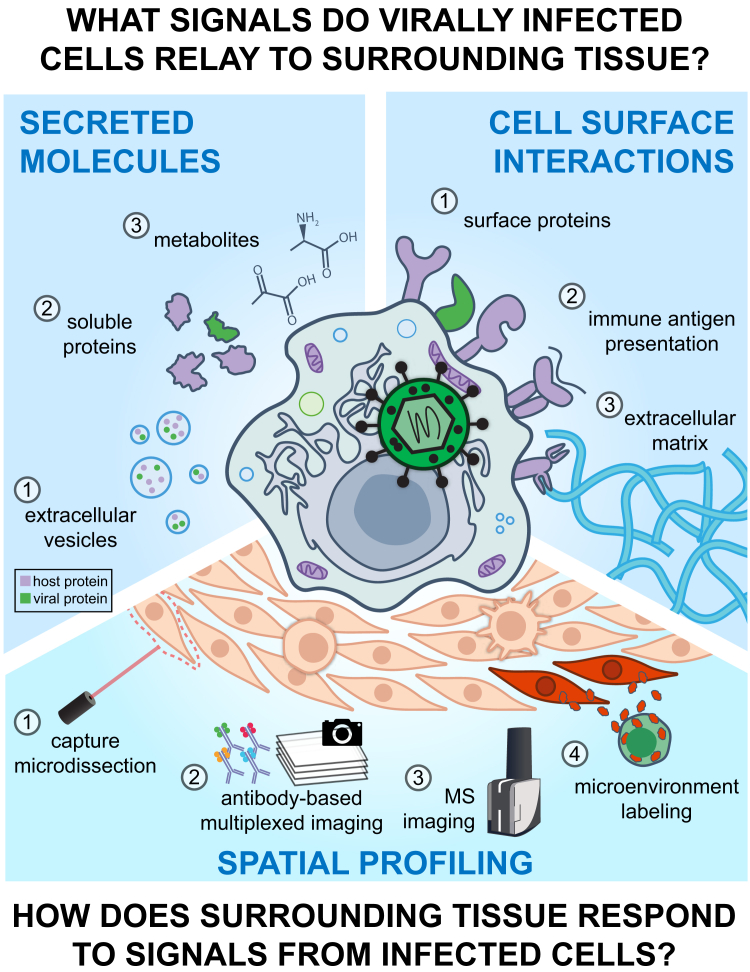
**Infected cells modulate their microenvironment in ways that are unique to the virus and infection site.** Many of the intercellular communication strategies employed by infected cells can be directly interrogated using proteomic tools, providing insight into the messages that infected cells relay to the surrounding tissue. Secreted metabolites and proteins, including viral proteins, potentiate diverse responses in recipient cells that have functional consequences for the outcome of infection. Similarly, alterations to protein–protein interactions at the cell surface between cells and cell–ECM can dictate infection outcomes. How cells in a virus microenvironment respond to signaling from infected cells is a function of their spatial proximity to sites of infection. Given this, spatial proteomic profiling methods represent powerful approaches towards uncovering how infection-induced modulation of the microenvironment promotes viral spread and/or pathogenesis.

To better understand these complex virus–host interactions, one must consider the myriad signaling mechanisms present within a virus microenvironment (VME) (Fig. 1). Among these intercellular communication mechanisms are those using secreted molecules, such as ions, metabolites, host and viral nucleic acids and proteins, virions, and EVs that contain diverse cargoes. Other communication mechanisms exploit interactions at the cell surface, involving the specific proteins expressed and their organization within the membrane, antigens that are presented to immune cells on MHC molecules, and cell–ECM contacts. Moreover, communication events also depend on the types of cells that are present in the microenvironment and their spatial organization in the tissue. Careful consideration of each of these aspects supports a nuanced view of how viral infection alters the surrounding uninfected tissue and how these changes enhance viral spread or clearance, dictating the outcome of pathogenesis.

Advancements in infection model systems, chemical labeling and physical isolation strategies, and multi- and spatial-omics techniques have enabled study of the VME in unprecedented detail. Given the central role of proteins in intercellular signaling processes, proteomic and adjacent mass spectrometry (MS)-based approaches have majorly contributed to interrogating these myriad intercellular interactions within a VME. Here, we introduce these advanced proteomic methods and explore their pivotal role in unraveling the multifaceted interactions between viruses and the host tissues they invade.

## Secreted Proteins and Metabolites

The molecules that infected cells secrete into their cellular microenvironments are crucial mediators of both proviral and antiviral intercellular communications, reflecting the tug-of-war between virus and host cell for control of the VME. For example, pro-inflammatory cytokines secreted from infected cells signal to surrounding cells to mount antiviral responses and recruit immune cells to the site of infection, while secreted viral mimics of anti-inflammatory cytokines can curb immune responses (25). In this section, we introduce the techniques used to measure the proteins, either soluble or contained within EVs, and metabolites that are secreted by infected cells and discuss insights gained into how viruses alter intercellular signaling pathways.

### Extracellular Vesicles

Many of the proteins secreted during viral infection can be released either as freely soluble factors (discussed below) or packaged into EVs. EVs are small membrane bilayer–bound particles released by virtually all cell types (reviewed in (26)). EVs contain a rich mixture of proteins, lipids, and nucleic acids that can be released into recipient cells, potentiating diverse signaling effects (27). Frequently classified based on their biogenesis and size, types of EVs include exosomes, derived from the endocytic pathway and ranging from 30 to 200 nm in diameter, and ectosomes, which bud directly from the plasma membrane, such as microvesicles (100–1000 nm) or apoptotic bodies (>1000 nm) (28). EV encapsulation can have distinct functional consequences, such as protection or concentration of factors that work cooperatively. For instance, the lipid bilayer of EVs can shield proteins from extracellular proteases and immune factors, potentially enhancing their stability and delivery to recipient cells (29). This protection may allow certain immune modulators or viral factors to persist longer, thereby altering local or systemic signaling dynamics. Conversely, freely soluble proteins diffuse more readily, enabling rapid communication with other cells in their environments (30). Thus, viruses may differentially exploit these pathways by leveraging EV-associated cargo for protected delivery of immunomodulatory proteins while simultaneously driving the secretion of soluble proteins to modulate the host response. Indeed, dynamics of EV secretion and their composition are profoundly altered during viral infections, with many viruses co-opting EV pathways to enhance their replication and evade immune detection (31, 32). Infection rewires host EV contents, including incorporation of viral proteins and viral RNAs (31, 32). Analyzing EVs released from infected cells reveals how their cargo changes in response to infection and provides insight into how EVs from infected cells can influence surrounding cells.

**Fig. 2:**
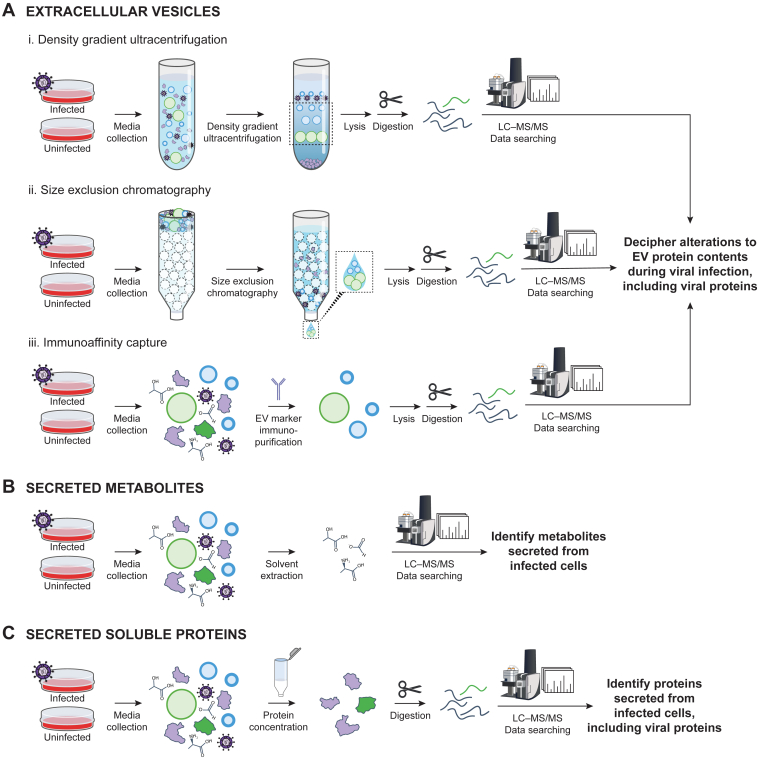
**Experimental techniques to decipher infection-induced alterations to secreted protein and metabolite profiles.***A*, measurement of proteins packaged within extracellular vesicles. Extracellular vesicles can be isolated from media conditioned by uninfected or infected cells *via* (i) density gradient ultracentrifugation, (ii) size-exclusion chromatography, or (iii) immunoaffinity capture prior to proteolytic digestion and LC-MS–based proteomic characterization. *B*, secreted metabolites can be profiled by enrichment from conditioned media *via* solvent extraction and subsequent metabolomics by LC-MS. *C*, quantification of secreted soluble proteins can be achieved by the concentration of protein from conditioned media by ultrafiltration before proteolytic digestion and LC-MS–based proteomic analysis.

The study of EVs during viral infection requires careful isolation to ensure a pure preparation prior to MS-based proteomics (33). We refer the reader to recent guidelines from the International Society for Extracellular Vesicles that can aid in experimental design (28) but highlight considerations useful for viral infection studies. Because complex media supplements (*e.g.*, serum) contain contaminating EVs, cells should be cultured in media that is serum-free or depleted of EVs. Once cell culture–conditioned media is obtained, several analytical techniques can be used to isolate EVs (reviewed in (34)). These techniques include (i) density gradient ultracentrifugation, which separates particles by density but can be harsh, (ii) size-exclusion chromatography, which is gentler, preserving vesicle integrity and function and separating particles by size using porous beads, and (iii) immunocapture, which is also gentle, purifying particles based on EV surface markers (*e.g.*, CD9, CD63, or CD81) and, thereby, requiring prior knowledge of such markers and risking missing unknown EV subpopulations (Fig. 2*A*). Since virus particles can share similar physiochemical properties with certain classes of EVs, careful consideration is required to avoid capturing virus particles in EV fractions. Multiple isolation strategies can be combined to improve confidence of EV separation from virus particles (35). For instance, one approach begins with bulk EV purification using polyethylene glycol, followed by column purification and affinity-based isolation using antibody-conjugated beads that target tetraspanin markers. These steps remove viral particles, while retaining EV-associated proteins and miRNAs (35), which is validated by measuring viral DNA content. Another method to validate that virus particles have been excluded from EV fractions involves the testing of these fractions for infectivity by viral titer (11).

Once EVs are isolated, validation of EV purity is essential to ensure the reliability of analyses. Several validation methods are available, including nanoparticle tracking analysis, which measures size distribution and concentration by tracking the Brownian motion of particles, confirming the presence of vesicles within the expected size range (36). Another method used is transmission electron microscopy that provides morphological assessment at high resolution, verifying vesicle integrity and size, and allowing direct visualization of EVs (37). Also used is the detection of known EV markers (*e.g.*, CD63, CD81, and Alix) in resulting proteomics data, which helps to verify the expected signatures of EVs (38). Flow cytometry also allows phenotyping of EV surface markers, with recent advances enabling the analysis of single EVs (39). The successful isolation and validation of EVs has enabled downstream analyses, such as MS, establishing myriad mechanisms in which viral and host EV pathways are altered during infection.

Virus-acquired mechanisms take advantage of EV biogenesis pathways to promote viral dissemination or modulate immune responses. For example, herpes simplex virus 1 (HSV-1) alters the EV population during infection (23, 40, 41, 42, 43). HSV-1–infected cells release EVs containing viral proteins, such as VP16, Us11, and the viral entry glycoprotein D, which can induce changes in the recipient cell to favor infection (23). Additionally, these EVs can carry host molecules, like the innate immune adaptor protein STING, which can influence the outcome of an infection in recipient cells (23, 41). The isolation of two EV subpopulations released during HSV-1 infection (CD63+ or ESCRT+) showed opposing functions: CD63+ EVs promote antiviral, while ESCRT+ EVs promote proviral responses (23). Other herpesviruses, such as human cytomegalovirus (HCMV), also modulate the host exosome biogenesis pathway for virion assembly and egress (10, 11).

Another herpesvirus, Epstein-Barr virus (EBV), has similarly acquired mechanisms to use the EV pathway (44, 45, 46, 47, 48). EBV's latent membrane protein 1 (LMP1) is incorporated into EVs and influences their formation through interactions with host proteins, like Syntenin-1 and Hrs, promoting the secretion of viral components that enhance cell proliferation and migration, contributing to oncogenesis (46). LMP1-containing EVs can activate signaling pathways in recipient cells, such as NF-κB and MAPK/ERK, leading to altered cellular behavior linked to the enhancement of EBV-associated cancers (45).

During SARS-CoV-2 infection, EVs have been shown to carry viral proteins and modulate the immune response (49, 50, 51, 52). EVs from SARS-CoV-2–infected cells contain viral proteins, like the nucleocapsid and spike proteins, and are enriched with integrins and immune signaling molecules, such as IL-6 and TGF-β, which may contribute to the cytokine storm observed in severe COVID-19 cases (49). In mild cases of COVID-19, patient EVs have higher levels of MHC-II, leading to antigen presentation to CD4^+^ T helper cells and more efficient immune activation (52). Another cytoplasmic single-stranded RNA virus, Rift Valley fever virus, similarly demonstrates the ability for EVs to also serve a protective role in host cells through the promotion of antiviral responses. EVs from Rift Valley fever virus–infected cells carry viral components that activate the RIG-I pathway, triggering a type I IFN response in recipient cells and enhancing autophagy, a protective mechanism against viral replication (53).

Hepatitis C virus (HCV) exploits EV pathways by incorporating viral RNA and proteins into EVs, facilitating transmission of these viral molecules to uninfected cells without detection by neutralizing antibodies (54, 55). HCV proteins, such as NS5A, interact with host apolipoprotein E in such vesicles, which is required for the efficient release of intraluminal vesicles containing HCV RNA (54). Similarly, in the case of hepatitis B virus (HBV), viral proteins like HBx are packaged into EVs, influencing the immune response and potentially aiding in immune evasion (56). Viral nucleic acids packaged into EVs during HBV infection are crucial mediators for intercellular communication, particularly between infected hepatocytes and cells of the innate immune system (57, 58).

HIV-1 secretes the viral protein Nef, which is packaged into EVs and released into the extracellular environment (15, 59, 60). Nef increases the abundance of lipid rafts in recipient cells, ultimately leading to phosphorylation of ERK1/2 and the secretion of pro-inflammatory cytokines (59). This secretion of Nef contributes to chronic immune activation in HIV-infected individuals, promoting disease progression and immune exhaustion (60).

EVs can play a critical role in modulating the host immune response even when originating from noninfectious cells, as is the case during varicella-zoster virus infection (18). Noninfectious varicella-zoster virus–infected cells secrete EVs that can modulate the immune response by delivering viral antigens and microRNAs to uninfected cells, potentially suppressing antiviral responses and facilitating immune evasion prior to viral spread (18). Zika virus (ZIKV) similarly alters the composition of EVs, which is relevant given its role in influencing the maternal–placental interface (17). The utilization of a highly translational model, rhesus macaque trophoblasts, demonstrated permissiveness to infection and altered EV mRNA, miRNA, and protein cargo, irrespective of productive infection (17).

Given their pivotal role in viral dissemination and immune modulation, EVs represent promising targets for therapeutic intervention. Blocking the secretion of EVs or modifying their content could potentially reduce viral spread and improve immune responses (61). For instance, inhibitors of exosome biogenesis, such as GW4869, have been shown to limit the transmission of viral particles or viral proteins between cells in the context of HCV, HIV, and HCMV infections (20, 60, 62). Furthermore, targeting specific viral or host proteins packaged into EVs, such as LMP1 in EBV or NS5A in HCV, could reduce the oncogenic and immunosuppressive effects of these vesicles (24, 63). Continued research into the specific roles of EVs in various viral infections will provide valuable insights into their therapeutic potential, offering new avenues for combating viral pathogenesis.

### Secreted Metabolites

In addition to traditional roles as molecular building blocks and intermediates in energy production, metabolites are increasingly recognized as intercellular signaling molecules (64). For example, secreted metabolites play important roles in tuning immune responses during many cancers (29). For several tumors, the specific composition of metabolites can condition an immunosuppressive microenvironment that prevents T cell activation and recruitment and promotes T cell exhaustion (30). Viral infection also extensively rewires cellular metabolism (65, 66), driving alterations to the profile of secreted metabolites and modulating the local microenvironment (67). Profiling metabolites secreted by infected cells therefore provides meaningful insight into the ways in which viruses influence their microenvironment.

MS-based metabolomics is a powerful, unbiased approach for the detection and measurement of metabolites secreted from infected cells. By comparing the metabolomic profile of media conditioned by infected *versus* uninfected cells, infection-induced changes to metabolite secretion can be identified. However, similar to the study of EVs, the presence of analyte (*i.e.*, metabolites) in cell culture media presents an analytical challenge (68). Not only do supplements, like serum, contain exogenous metabolites, but so does the basal media itself, though, for practical cell culture considerations, this is unavoidable. A typical workflow therefore involves exchanging growth media for serum-free medium, then allowing time for metabolite secretion before collecting. Once conditioned media is obtained, metabolic processes are quenched and metabolites are extracted, frequently in one step by ice-cold solvent extraction, before performing metabolomic LC–MS analysis (reviewed previously (69, 70); Fig. 2*B*). MS acquisition in both positive and negative ion modes can enhance metabolome coverage (71).

The metabolic reprogramming of infected cells is a common theme among viruses, as it provides energy and biosynthetic precursors necessary for viral replication (72). During infection, host cells release a range of metabolites, such as lactate, kynurenine, and polyamines, which can have both immunosuppressive and immune-stimulatory effects (73, 74, 75). Lactate, a byproduct of glycolysis, is often secreted in large quantities during viral infections as a result of enhanced aerobic glycolysis or the "Warburg effect," a hallmark of metabolic reprogramming in infected cells (76). Elevated extracellular lactate has been shown to promote an immunosuppressive microenvironment by inhibiting cytotoxic T cell and NK cell activity, while simultaneously favoring regulatory T cell differentiation (77).

Another key metabolite involved in intercellular communication during viral infection is kynurenine, a product of tryptophan catabolism through the indoleamine 2,3-dioxygenase pathway (78). Viral infections, such as those caused by HIV, HBV, SARS-CoV-2, and HCMV lead to increased kynurenine levels (75, 79, 80, 81). Kynurenine acts as an immunosuppressive molecule by activating the aryl hydrocarbon receptor on immune cells, which downregulates inflammatory responses and promotes immune tolerance (82). This pathway is exploited by viruses to evade host immunity, as evidenced by the correlation between high kynurenine levels and disease progression in HIV-infected patients (80).

Polyamines, a group of metabolites derived from amino acid metabolism, are also critical players in virus spread (83). They are essential for stabilizing DNA, RNA, and ribosomes and have been implicated in the replication of numerous viruses, including ZIKV and dengue virus (84). For instance, ZIKV infection has been shown to upregulate polyamine synthesis, and inhibition of polyamine production reduces viral replication, highlighting the potential of targeting polyamine metabolism as a therapeutic strategy (85).

The secreted metabolites released during viral infections can also act as damage-associated molecular patterns, signaling tissue stress and activating innate immune pathways (86). Adenosine, a purine metabolite released during cellular stress and tissue damage, serves as a signaling molecule that can suppress the immune response by engaging adenosine receptors on immune cells. The adenosine pathway is linked to dysregulation during HIV infection and is implicated in HIV-associated chronic inflammation (87).

Overall, the complex interplay between viruses and host metabolic pathways underscores the importance of secreted metabolites in viral pathogenesis. Understanding these interactions can reveal new therapeutic targets for modulating immune responses and inhibiting viral replication. With continued advancement of metabolomics technologies, the role of secreted metabolites in intercellular communication during viral infection will be further elucidated, providing a deeper understanding of host–pathogen interactions.

### Secreted Soluble Proteins

Secreted soluble proteins are among the most widely appreciated intercellular signaling molecules, governing many aspects of development, cell growth and differentiation, and immunity (4, 88). Through diffusion outwards from the secreting cell, soluble proteins can reach and potentiate a response in nearby cells (*i.e.*, paracrine signaling), as well as far away cells (*i.e.*, endocrine signaling). Both paracrine and endocrine mechanisms are engaged during viral infection to mount an immune response, while virus infection–driven changes can dampen or counteract these signals (25, 89). In addition to contributing to immune modulation, secreted soluble proteins play multifaceted roles in viral infections, viral pathogenesis, and tissue remodeling. Given this exciting interplay and the importance of secreted soluble proteins in intercellular signaling, profiling proteins secreted by infected cells provides information about cell communication with their microenvironment. Understanding how viruses manipulate the secretion of these proteins provides critical insights into viral strategies for persistence and immune evasion and highlights potential targets for therapeutic intervention in a wide range of viral diseases.

MS-based secretome analyses provide an unbiased means to detect secreted proteins from cultured infected cells (reviewed previously (90, 91)). As with the approaches mentioned above, analysis of soluble proteins typically involves culturing cells in serum-free media to avoid contaminating exogenous proteins from serum. Because the concentration of secreted proteins can be low, protein from collected conditioned media is frequently concentrated using filtering devices prior to proteolytic digestion and LC–MS (92). In addition to MS-based approaches, targeted approaches, like the Luminex multiplex assay or ELISA, can be used for the quantification of defined protein panels, such as cytokines (93).

A critical class of secreted soluble proteins during viral infection is cytokines. These proteins are central to coordinating the immune response, with infection-induced modulation of cytokine signaling being linked to resulting pathologies (94). SARS-CoV-2 infection is a notable example, where the dysregulated secretion of pro-inflammatory cytokines like IL-6, IL-1β, and TNF-α contributes to the cytokine storm (95). This excessive immune activation results in widespread inflammation and tissue damage, particularly in the lungs, and is associated with poor outcomes (96). Comprehensive mapping of SARS-CoV-2 protein binding to human RNAs reveals a potential mechanism by which type I IFN responses are inhibited. Two SARS-CoV-2 proteins, NSP8 and NSP9, bind to the RNA component of the signal recognition particle and restrict protein trafficking to the membrane, a crucial step for the secretion of most cytokines (97).

HBV and HCV exploit the secretion of soluble immunomodulatory proteins to create a more favorable environment for viral persistence. HBV causes infected cells to secrete large amounts of hepatitis B surface antigen, which acts as a decoy, binding to antibodies and preventing the immune system from targeting actual virus (98) and facilitating chronic infection (99). A mechanism ensuring viral persistence was also reported for HCV-infected cells, which secrete immunosuppressive cytokines such as IL-10 to dampen the host's antiviral response and allow the virus to persist within the liver for extended periods (100). The secretion of these cytokines effectively reduces the cytotoxic activity of T cells and NK cells, hindering the clearance of infected cells (101).

Herpesviruses also use secreted soluble proteins for immune evasion. EBV infection leads to the secretion of viral proteins, such as viral IL-10, which mimics human IL-10 and downregulates the host immune response by inhibiting the activity of APCs and reducing the secretion of pro-inflammatory cytokines (102). This enables EBV to evade immune surveillance and maintain latency in infected cells, contributing to its oncogenic potential (102). HSV-1 also secretes viral proteins that interact with host immune mechanisms, such as glycoproteins E and I, which form a complex that binds to the Fc region of IgG antibodies, interfering with antibody-mediated immune responses (103). These proteins help the virus evade immune detection and prevent effective clearance by neutralizing antibodies.

In addition to immune modulation, secreted soluble proteins during viral infections have profound effects on the local tissue microenvironment. For instance, ZIKV infection in placental trophoblasts leads to the secretion of various pro-inflammatory and profibrotic proteins, which can alter tissue architecture and contribute to placental dysfunction (104). These secreted proteins include matrix metalloproteinases and growth factors, which degrade the ECM and promote tissue remodeling, potentially facilitating viral dissemination (105, 106).

Advances in 3D tissue culture techniques have facilitated the study of intercellular communication during viral infection in more physiologically relevant systems (107). This has been relevant to the study of secreted proteins, as illustrated by the human Gut-on-a-Chip microfluidic device that enables culturing intestinal epithelium under physiological fluid flow (108). This model revealed a polarized release of cytokines to the luminal side of the epithelium, as well as a gradient of cytopathic effects resulting from directional fluid flow (108). Various organoid models have also identified that infected cells are preferentially extruded into the fluid-filled organoid lumen, which may represent both proviral (109) and prohost (110) responses.

The modulation of soluble protein secretion by viruses presents another avenue for therapeutic intervention. Targeting key secreted proteins, such as pro-inflammatory cytokines or viral decoy proteins, could help restore immune function and prevent tissue damage during infection. For example, therapeutic approaches aimed at neutralizing IL-6 during COVID-19 have shown promise in reducing the severity of cytokine storms and improving clinical outcomes (111, 112). Similarly, inhibiting the secretion of viral proteins like hepatitis B surface antigen in HBV infections could enhance the effectiveness of antiviral therapies by allowing the immune system to better recognize and clear infected cells (113, 114).

## Interactions at the Cell Surface

The cell surface provides a specialized interface for intercellular communication. Given the closeness of contact required, interactions at the cell surface between cells or cell–ECM are comparatively extensive. For instance, the interaction of an infected cell presenting a viral antigen *via* MHC-I and CD8+ T cell’s T cell receptor forms an immune synapse, the tightness of which underlies the ability of the T cell to engage in further signaling and enact its cytotoxic activity (114). Interactions between cells and the ECM often control primary cell processes like adhesion and migration and drive many viral pathologies.

### Cell Surface Profiling

Viruses are potent modulators of protein abundances at the cell surface, for example, as part of mechanisms to evade host immune surveillance (115, 116). Profiling the proteins displayed on the cell surface by MS provides broad insights into the interactions an infected cell can engage in with other cells or the ECM.

**Fig. 3:**
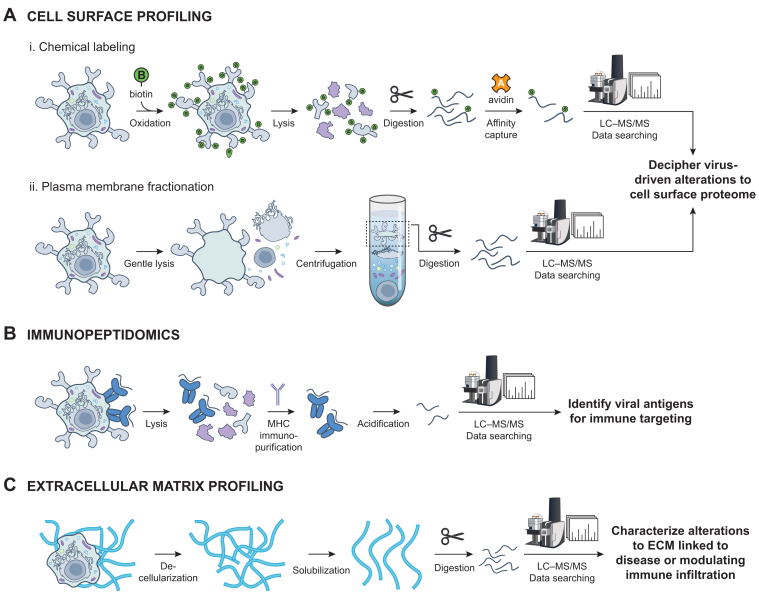
**Proteomic tools to probe infection-induced changes to cell surface interactions.***A*, measurement of proteins expressed on the cell surface. Cell surface proteins can be enriched by (i) chemical labeling of extracellularly facing proteins with an affinity probe or (ii) subcellular fractionation, collecting the plasma membrane fraction, prior to proteolytic digestion and LC-MS–based proteomic characterization. *B*, detection of antigen peptides presented by MHC molecules can be achieved by immunopurification of MHC molecules prior to LC–MS analysis of antigen peptides released by acidification. *C*, extracellular matrix proteins can be profiled by removal of cells, followed by solubilization and proteolytic digestion before LC-MS–based proteomics.

Owing to their high hydrophobicity and low relative abundance, cell surface proteins are often underrepresented in whole cell proteomics. Accordingly, specialized enrichment strategies have been developed (reviewed previously (117, 118, 119)) to enhance coverage and boost confidence that identified proteins are displayed on the cell surface and not elsewhere within the cell. Two enrichment methods are frequently used: (i) tagging cell surface proteins for affinity capture or (ii) subcellular fractionation *via* differential centrifugation to isolate the plasma membrane (Fig. 3*A*). Tagging of the cell surface can be achieved by treating cells with membrane impermeable biotinylation reagents, such as Sulfo-NHS-SS-biotin (120), to label surface-exposed primary amines of lysine and *N-*terminus. Affinity capture of biotinylated peptides following proteolytic digestion enriches cell surface–exposed proteins. Subcellular fractionation, often part of a broader cell fractionation effort, not only informs on intracellular organization but can also contextualize changes occurring at the cell surface. The imprecise delineations between organelle fractions presents a challenge, but computational strategies, including machine learning–based approaches, aimed to increase the confidence of protein organelle assignments are available (121, 122, 123). Temporal dynamics can also be captured by performing profiling experiments at multiple timepoints of infection, thus providing additional information about surfaceome regulation. Cell surface profiling strategies have been employed across diverse infection contexts and have provided extensive evidence for the capacity of viruses to alter intercellular interactions and, in particular, evade immune surveillance.

Cell surface profiling during HCMV infection has identified hundreds of modulated cell surface proteins (124, 125, 126, 127, 128, 129, 130). When considering cell–cell communication, emphasis has been placed on immunomodulatory components, such as the MHC-I. HCMV downregulates cell surface expression of MHC-I to mitigate T cell–mediated cytotoxicity. Downregulation is achieved *via* degradation, as well as sequestration of MHC-I in the ER by HCMV proteins (131). ER sequestration of MHC-I has been captured in studies profiling intracellular compartments in addition to the cell surface (124, 125, 128). Cell surface proteins previously unknown to be regulated by HCMV have also been identified by cell surface profiling studies, including protocadherins, ephrins, plexins, and immunoglobulins (124). The importance of these alterations to immune evasion has been demonstrated by NK and T cell degranulation assays. Additionally, several identified HCMV-modulated proteins have been proposed to have novel immunomodulatory roles. The C-type lectin CLEC1a was downregulated by HCMV infection and may be an activating NK ligand, while the immunoglobulin family member CEACAM-1 was upregulated and may be a negative regulator of cytotoxic T cell–mediated lysis (124). These findings highlight the potential for cell surface profiling during viral infection to uncover protein functions. In addition to immunomodulatory proteins, HCMV also alters the expression of cell junction proteins (124, 125), as well as of proteins involved in cell adhesion and migration (125, 127, 129). Altogether, these alterations to the cell surface during HCMV infection highlight several of the strategies employed to evade the immune system and suggest changes to cell motility.

Cell surface profiling has similarly revealed mechanisms of immune evasion employed by HSV-1 (132), EBV (133), and HIV-1 (134, 135, 136, 137). During HSV-1 infection, further evidence was provided for intracellular sequestration of MHC-I, and downregulations of TLR2 and IL18R immune receptors were discovered (132). These changes suggest likely viral strategies to decrease inflammation and immune activation. In two B cell models of EBV lytic replication, the B cell receptor complex was found to be downregulated (133). As the B cell receptor complex mediates B cell activation, this may represent a viral strategy to reduce inflammation. In a T cell model of HIV-1 infection, >100 plasma membrane proteins were found to be downregulated, including proteins involved in T cell activation and cell adhesion (*e.g.*, MHC-I, CD28, and CD53), as well as the transmembrane transporter SNAT1. A metabolomic profiling approach revealed alanine as a SNAT1 substrate in CD4+ T cells and demonstrated that extracellular alanine is crucial for T cell mitogenesis (134). Therefore, HIV-1–mediated downregulation of SNAT1 negatively regulates immune cell activation.

While emphasis has been placed on the modulation of host proteins, due to their better characterized functions, details are also emerging about the capacity of viral proteins displayed at the cell surface to engage in intercellular communication. Indeed, viral proteins are also captured in cell surface profiling studies (124, 125, 126, 127, 128, 129, 130, 132, 133, 134, 135, 136, 137, 138).

In addition to the cell surface profiling methods mentioned above, approaches used for characterizing protein–protein interactions (PPI) have also proven valuable for capturing changes at the cell surface. For instance, thermal proteome coaggregation profiling, a method for studying global PPIs, uncovered CD63-dependent internalization of the HCMV coreceptor ITGB1 (139). Additionally, characterization of PPIs formed between cells or at the cell–ECM interface can provide insights into intercellular communications and molecular mechanisms employed by viruses to enhance their replication and spread. A suite of proteomic technologies exists to label these transcellular protein interactions (140, 141, 142, 143, 144, 145, 146); however, while several of these approaches have been used to decipher specific viral entry mechanisms (145, 147, 148, 149, 150, 151, 152, 153), to our knowledge, they have yet to be applied to probe cell–cell or cell–ECM interactions in the context of a viral infection. It is tempting to propose, for example, that probing the immune synapse between an infected cell and a T cell would uncover additional mechanisms of immune evasion employed by viruses.

### Immunopeptidomics

Antigen presentation is a primary host defense mechanism against pathogens, enabling the detection and removal of infected cells by the immune system (reviewed in (154)). Within an infected cell, viral proteins are degraded into small peptide fragments (∼8–11 amino acids) and loaded onto MHC molecules for presentation to T cells. T cell recognition of viral antigens prompts targeting of infected cells for destruction. Despite numerous virus-evolved strategies to suppress antigen presentation, recognition and killing of infected cells displaying viral antigens represents a key mechanism of viral clearance (155), making antigen presentation *via* MHC molecules a critical interface for communication between infected and immune cells during viral infection.

MS-based immunopeptidomics has emerged as a powerful tool for the unbiased detection and measurement of viral antigens displayed on MHC molecules (reviewed previously (156, 157)). Prior to LC–MS/MS, MHC-bound peptides must be enriched (158, 159), which is required due to the small size, high sequence diversity, and low abundance of MHC peptides. Purification is typically achieved by immunopurifying MHC molecules, then acidifying to release MHC-bound peptides (Fig. 3*B*). Despite the relative simplicity of the workflow, immunopeptidomic experiments can be complicated to perform owing to several factors, including (i) the requirement for large amounts of starting material given the low abundances of immunopeptides, (ii) the inability to detect all MHC peptides due to incompatibilities with reverse-phase chromatography and/or electrospray ionization, (iii) the large data searching space due to diverse peptide cleavage sites and PTMs, and (iv) the contribution of noncanonical open reading frames (ORFs) to the MHC-restricted peptidome. Nonetheless, recent advances in immunopeptidomics sample preparation, MS acquisition, and data searching methods as well as integrations with genomic technologies have taken significant steps to overcome these challenges (160, 161, 162, 163, 164) and, together, have contributed to the discovery of thousands of viral antigens across diverse viral infection contexts.

During the past 5 years, there has been intensive effort to profile the immunopeptidome during coronavirus infections (165, 166, 167, 168, 169, 170, 171). As a result, viral antigens have been identified deriving from every OC43 (a seasonal coronavirus) protein (165) and from most SARS-CoV-2 proteins across both MHC-I (166, 167, 169) and MHC-II classes (167, 168, 171). Comparing peptidomes across MHC classes revealed that MHC-I and MHC-II pathways target distinct viral proteins. Structural proteins predominantly contribute to the MHC-II peptidome, while nonstructural and noncanonical proteins largely comprise the MHC-I peptidome (171). Evidence also points to an increased contribution of viral proteins that are expressed early in infection to the MHC-I repertoire (169). The SARS-CoV-2 spike (S) protein contributes >200 unique peptides to the MHC-II peptidome in dendritic cells. Interestingly, while the S protein is heavily glycosylated on the virus surface, many of the MHC-II peptides deriving from glycosylation sites were not, potentially indicating a mechanism for trimming of glycan residues during antigen processing (168).

An exciting aspect of immunopeptidomic studies is the ability to demonstrate the importance of identified viral antigens by testing their immunogenicity using functional assays that measure CD4+ and CD8+ T cell responses (165, 167, 169). In one such study, MHC-I peptides deriving from noncanonical ORFs were found to elicit T cell responses that exceeded those from most canonical peptides in humanized mice and COVID-19 patients (169), highlighting the value of considering peptides from noncanonical viral ORFs in immunopeptidomic studies. Ribosome profiling represents a powerful approach for uncovering such noncanonical viral ORFs (161, 164, 172). Application of a recently developed ribosome profiling method, termed Massively Parallel Ribosome Profiling, led to the discovery of >5000 ORFs from almost 700 different human-associated viruses of which >4000 were noncanonical. Searching preexisting immunopeptidomics spectra against this database demonstrates its utility, as it expanded the number of peptides matched across a variety of viral infections (161).

Immunopeptidomic profiling has provided key functional insights into immune responses across a range of other viral infections (173, 174, 175, 176, 177, 178, 179, 180, 181, 182, 183, 184). For instance, the oncovirus human papilloma virus (HPV) has been shown to restrict the repertoire of antigens presented on MHC-I molecules *via* HPV oncoprotein E5 (174). Despite this, 11 MHC-I peptides from HPV oncoproteins E6 and E7 were discovered using a sensitive, targeted MS3 approach and demonstrated to elicit CD8+ T cell reactivity. Several peptides that were identified using untargeted DDA were not found using the targeted approach from the same sample, highlighting the potential for false positive identifications in immunopeptidomics studies (173). In addition to targeted MS approaches, the use of multiple cell models can also increase confidence for identified antigens. Comparison of MHC-I and -II peptidomes across multiple models of influenza infection revealed differential repertoires, with the influenza matrix 1 protein being the most frequent source of MHC-I and MHC-II peptides across infection models (176). Influenza antigens can also arise from understudied sources, such as the M-SL9 antigen (175). M-SL9 maps to a previously unannotated ORF in the influenza genome and was discovered by immunopeptidomic profiling of MHC-E peptides, a nonclassical MHC molecule. M-SL9 was demonstrated to drive an effective T cell response and strong differentiation into tissue-resident T cells (175), further illustrating the value of considering nonclassical MHC molecules and viral ORFs in immunopeptidomic studies.

Given the continuous advancements in MS platforms and computational analyses, the immunopeptidomics field is rapidly evolving, being poised to decipher specific viral antigens presented by infected cells and to clarify principles of immune recognition.

### Extracellular Matrix Profiling

The ECM is a dynamic network of structural proteins, proteoglycans, glycosaminoglycans, and mucus whose precise molecular, physical, and mechanical properties control cell adhesion, migration, and signaling. As such, the ECM is a critical component regulating intercellular communication. During viral infection, the ECM undergoes significant remodeling (185), leading to alterations that can facilitate viral spread (186), immune cell infiltration (187, 188, 189), or tissue damage (190, 191). Accordingly, there is growing appreciation for the interplay between ECM biology, immune function, and pathobiology during viral infection.

Bottom-up MS-based proteomics has been one of the preferred methods for molecular characterization of ECM (reviewed previously (192)). As many ECM components are highly insoluble given their large size, extensive PTMs, and/or tendency to assemble into higher-order molecular structures, their analysis benefit from specialized enrichment strategies. Enrichment protocols remove cellular material (decellularization), often using chelating agents and mild detergents, prior to the enhancement of ECM protein solubility using harsh detergents, chaotropic agents, and/or protein deglycosylation prior to proteolytic digestion (193, 194, 195, 196, 197) (Fig. 3*C*). For downstream database searching, the inclusion of modifications that are unconventional for typical global proteomic analyses (*e.g.*, lysine and proline oxidation) improves coverage (198). While it is advantageous to enrich for ECM components (199, 200, 201), global proteomic analyses can still yield valuable insights into ECM composition (186, 191, 202, 203, 204). Thus, both approaches represent powerful tools for uncovering ECM remodeling and regulation during viral infection.

Proteomic profiling of ECM has played a key role in linking ECM dysregulation to congenital Zika syndrome (186, 202, 205). ECM proteins involved in structure and organization, including fibronectin, were found to be altered during placental ZIKV infection. Upregulation of fibronectin was validated immunohistochemically and is suggested to promote restructuring of ECM, affecting the tissue’s integrity and possibly enhancing dissemination of the virus from mother to fetus (186). In addition to a potential role supporting ZIKV perinatal transmission, dysregulation of ECM also appears to support congenital Zika syndrome pathology itself. Reduced expression of collagens, but increased expression of adhesion factors, in postmortem brains of neonates with congenital Zika syndrome was identified at both the RNA and protein levels and might contribute to microcephaly and neuronal migration disorders attributed to congenital ZIKV infection (202).

Analyses performed during influenza infection point to a similar capacity for influenza virus to promote spread and pathology by disrupting ECM regulation. Influenza A infection of the airway epithelium induces a cystic fibrosis–like pathophysiology that increases susceptibility of the tissue to secondary infection by *S. pneumoniae*. Acidification of the airway surface liquid at the ECM is thought to be a primary mechanism for this increased susceptibility, for which proteomic profiling provides important evidence (22). Depletion of multiple collagen types, basement membrane-associated complexes, basal-cell-adhesion molecules, glycoproteins, and proteoglycans was discovered and validated by light and scanning electron microscopy in mouse lung infected with H1N1 influenza virus. Interestingly, many of these components are known substrates of the ECM remodeling enzyme MT1-MMP, which is strongly upregulated during influenza infection. Inhibition of MT1-MMP activity protected lung tissue integrity during infection without impacting immune response (200). Upregulation of matrix metalloproteinase family proteins and associated disruption of ECM integrity was also demonstrated for Newcastle disease virus (201).

Given the important insights gained from profiling extracellular matrix proteins, the further implementation of other methods, such as glycoproteomic profiling (206) and imaging MS (207) of ECM, promise to deepen the understanding of ECM biology surrounding infection sites.

## Spatial Profiling

How a cell responds to the myriad messages derived from infected cells is a function of its spatial positioning relative to a site of infection. By preserving spatial contexts, spatial profiling approaches offer a holistic view of how viruses modulate the VME during infection, providing information about alterations to cellular composition of a tissue, the spatial organization of cells within that tissue, where viral replication sites are, the extent of immune infiltration, and local concentrations of biomolecules.

### Tissue Dissection Mass Spectrometry

**Fig. 4:**
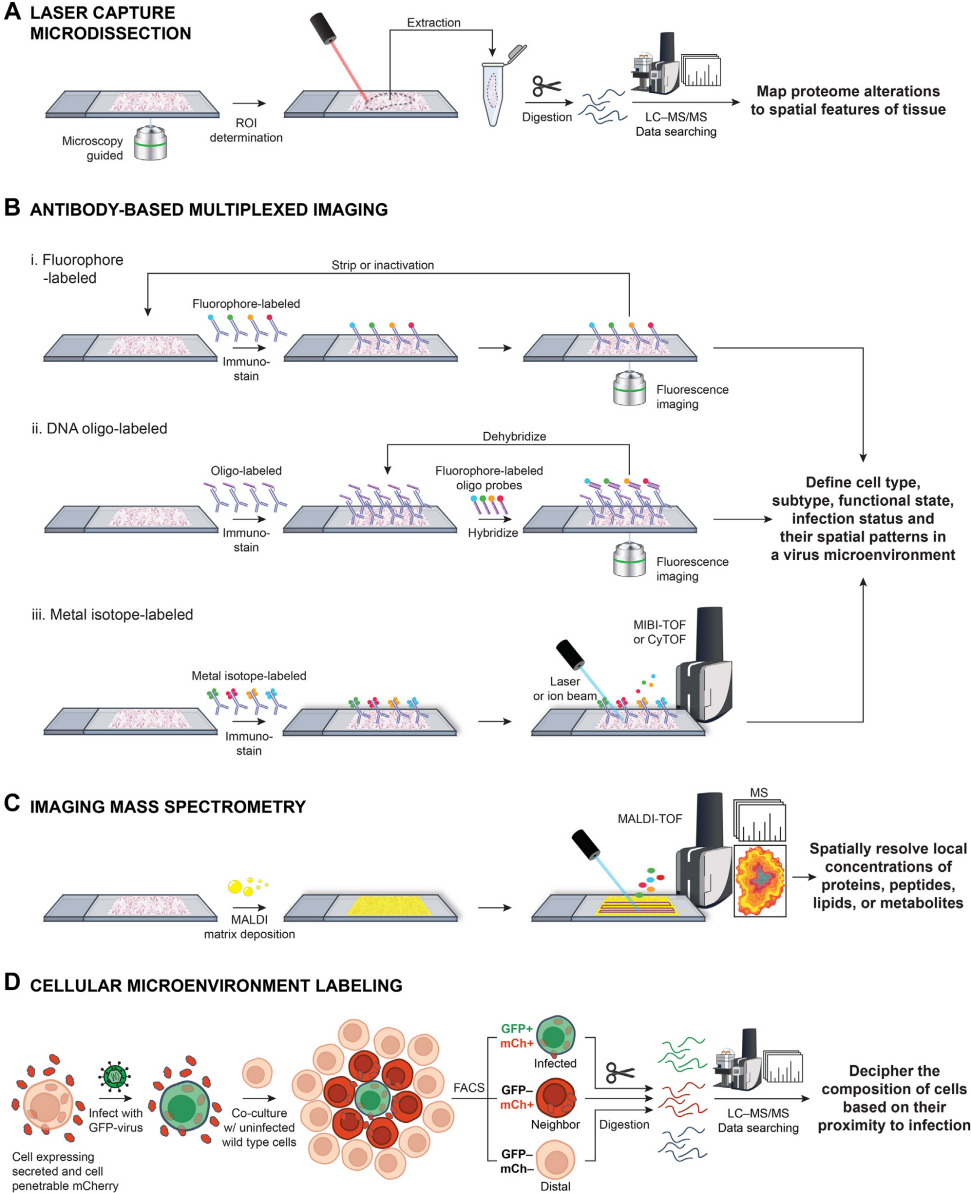
**Spatial proteomic approaches to characterize infection-induced alterations to the microenvironment.***A*, tissue dissection by microscopy-guided laser capture microdissection enables capture of tissue subsections prior to proteolytic digestion and LC-MS–based proteomic characterization. *B*, spatially resolved antibody-based measurement of proteins in a tissue. Selected proteins can be immunostained using (i) fluorophore- or (ii) DNA oligo-labeled antibodies and detected using fluorescence imaging or, alternatively, using (iii) metal isotype-labeled antibodies with detection by MS. *C*, unbiased, spatially resolved measurement of proteins, peptides, lipids, or metabolites in a tissue can be achieved by depositing a chemical matrix on a tissue slice prior to raster MALDI-MS. *D*, deciphering of alterations to proteomes of cells in a virus microenvironment based on spatial proximity to infected cells is enabled by proximity labeling *via* a secreted and cell penetrable red fluorescent protein followed by cell sorting and MS-based proteomic analysis.

The most conceptually straightforward method for preserving spatial context in MS-based proteomic experiments involves tissue dissection. Dissection by scalpel or laser capture microdissection (LCM) (reviewed in (208)) enables isolation of specific regions of interest, down to the subcellular level, for proteomic analysis. When performed in conjunction with histological examinations or other imaging methods, distinct regions can be delineated and collected based on a desired characteristic or set of characteristics. Some prior knowledge of the morphology or molecular characteristics of those regions is therefore required, slightly hindering application as a discovery tool. LCM is the preferred dissection strategy due to its speed, precision, and capacities for automation and integration with imaging data. For example, a recent study applied LCM in a semi-automated manner to capture hundreds of subcellular regions from hepatocytes in liver tissue using artificial intelligence to guide cell segmentation (209). To perform LCM, a tissue slice is mounted on a slide where it is stained and imaged before predefined sections are cut with a laser and automatically extracted to a tube or well-plate (Fig. 4*A*). Tissue dissection facilitates sampling of distinct tissue sections, thereby enabling investigations of the microenvironment during viral infection.

Tissue dissection MS has advanced our understanding of the unique pathobiologies of viruses such as HPV (210, 211) and SARS-CoV-2 (212, 213). Profiling of neoplastic epithelial cells isolated from oral squamous cell carcinoma tumors by LCM revealed a distinct microenvironment for HPV-positive tumors (210). Compared to HPV-negative tumors, the HPV-positive tumor microenvironment was characterized by a lower proportion of M1 macrophages and dendritic cells, but heightened innate immune signaling, including increased expression of S100A8, several pattern recognition receptors, and NFκB responses (210). These alterations were associated with poorer survival outcomes and underscore the capacity of HPV to modulate immune responses within the tumor microenvironment. During SARS-CoV-2 infection, tissue dissection MS illustrated the impact of cardiac anatomy on infection outcomes. Spatially resolved proteome mapping of postmortem hearts from COVID-19 patients indicated the left atrium to be more susceptible to SARS-CoV-2 infection and associated inflammatory injury than other heart regions (213). This region-specific vulnerability may be attributed to the left atrium’s enhanced connectivity to the virus and cytokine storm originating from the lung. Collectively, these studies exemplify the potential of tissue dissection MS to resolve how viruses modulate their microenvironments and cause disease.

### Antibody-Based Multiplexed Protein Imaging

The VME comprises a variety of cell types (*e.g.*, epithelial cells, fibroblasts, immune cells) that exist in different functional states (*e.g.*, infected or uninfected, activated, or exhausted T cells) and are spatially organized within a tissue. Defining these cell types, subtypes, and functional states while measuring their spatial patterns *in situ* in infected tissue offers invaluable insights about the mechanisms by which viruses can spread and cause disease. Amazingly, this level of detail can be captured in a single multiplexed protein imaging experiment.

Protein imaging predominantly relies on antibodies for the detection of predefined analytes. But whereas a typical fluorescence imaging experiment is limited to detection of just three or four proteins due to spectral overlap of fluorophores, multiplexing by iterative staining and/or use of clever antibody labeling and detection schemes enables imaging of >60 proteins in the same tissue at single cell resolution.

Methods for multiplexed protein imaging vary based on the mode of antibody tagging used (*i.e.*, fluorophore, metal isotope tag, oligonucleotide barcode) and the analytical method employed (*i.e.*, spectroscopy, MS) (reviewed previously (214, 215, 216)). The most straightforward approach involves iterative staining and imaging with fluorescently tagged antibodies: (1) immunolabeling of the tissue sample with a small subset of fluorescently tagged antibodies, (2) fluorescence imaging, (3) inactivation of fluorophores or stripping away of antibodies, and repeat (Fig. 4*B*i). Antibodies tagged with oligonucleotides can be used instead, sidestepping the need to perform multiple immunolabeling steps: (1) immunolabeling with full panel of oligonucleotide-tagged antibodies, (2) binding of a subset of oligonucleotide-tagged antibodies with complementary oligonucleotide probes that are fluorescently tagged, (3) fluorescence imaging, (4) inactivation of fluorophores or unbinding of oligonucleotide probes, and repeat steps 2 to 4 (Fig. 4*B*ii). Alternatively, MS can be used as a readout by labeling antibodies with ionizable metal isotope mass tags. Compared to immunofluorescence (IF) imaging, the low background and high resolving power of MS allow for simultaneous detection of >40 analytes in a single cycle. The most widely adopted antibody-based MS imaging methods are imaging mass cytometry (217) and multiplex ion beam imaging (218) (Fig. 4*B*iii).

An essential aspect of multiplexed imaging studies is antibody panel design (219). The panel should be designed so that all cell types, subtypes, and functional states of interest are labeled and, as such, requires prior knowledge of delineating factors. Many studies focus such panels on identifying immune subsets and their activation states, which are well studied, have commercially available antibodies, and are interesting given the critical roles that immune cells play in shaping the VME. It is additionally useful to include viral markers in studies of the VME to distinguish infected and uninfected cells. Panel design can also incorporate data-driven approaches. A recent study used scRNA-seq data to identify markers that can distinguish previously unknown macrophage subtypes (220).

Insight into the mechanisms employed by EBV to condition an immunosuppressive tumor microenvironment has been gathered using multiplexed imaging approaches. In EBV-positive classic Hodgkin’s lymphoma tumors, multiplexed IF imaging revealed a link between EBV-positive tumor cell MHC-I expression and a cell neighborhood rich in macrophages and CD8+ T cells, indicating an activated antiviral immune response (221). Profiling by multiplex ion beam imaging-TOF, however, showed that these recruited CD8+ T cells exhibit signatures of a terminally exhaustive state as a function of their proximity to EBV-positive tumor cells, suggesting a mechanism by which EBV counteracts an activated immune response by immunomodulating nearby T cells into a terminally exhaustive state (222). A different mechanism of immune suppression has been proposed in EBV-positive epithelial cancers. Multiplexed IF imaging integrated with spatial transcriptomics showed that EBV-infected cancer cells secrete CCL5 and CSF-1 cytokines, which recruit anti-inflammatory M2c-like macrophages to the tumor microenvironment (223). M2-macrophages, in contrast to pro-inflammatory M1 macrophages, exhibit immunosuppressive effects, contributing to matrix remodeling and favoring tumor growth (224).

An integrated DNA, RNA, and protein imaging approach termed protein and nucleic acid *in situ* imaging (225) uncovered a similar mechanism employed by simian immunodeficiency virus (SIV) (226). In lymphoid tissue from SIV-infected nonhuman primates, SIV infection induced IL-10 expression in B cells, promoting M2 macrophage polarization and contributing to an immunosuppressive VME (226).

By contrast, SARS-CoV-2 establishes a VME that is hyperinflammatory and is associated with tissue damage. Multiplexed multi-omic imaging of postmortem lung tissue from human and a nonhuman primate model demonstrated that SARS-CoV-2 infects primarily alveolar epithelial cells in the lung and that its VME is characterized by high levels of IFN-α, IL-6, and immune infiltration (227, 228, 229). Imaging mass cytometry showed that severity of lung damage is associated with increasing numbers of fibroblasts and mesenchymal cells and increased spatial proximity between them (228). Multiplexed IF imaging using an iterative fluorophore-labeled Ab staining and photobleaching method termed multi-epitope-ligand cartography (230), combined with spatial transcriptomics, points to upregulations in CCL21 and CCL18 chemokines as key factors driving endothelial-to-mesenchymal transition in the microenvironment. This finding can explain the identified increase in mesenchymal cells and promoting tissue fibrosis (231). These mechanisms together support a dysregulated immune response to SARS-CoV-2 infection that promotes extensive tissue remodeling that is active even beyond resolution of the infection.

The inflammatory effects of SARS-CoV-2 infection extend beyond just the lung. A multi-omic approach, including multiplexed IF profiling by multi-epitope-ligand cartography, of postmortem brain tissue from COVID-19 patients revealed that an inflammatory type I IFN response was present in central nervous system (CNS) tissue even after clearance of the initial infection (232). This study provides evidence for systemic inflammation induced during acute SARS-CoV-2 infection perturbing CNS homeostasis and possibly explaining some of the neurological symptoms associated with COVID-19. Systemic inflammation caused by viral infection is also associated with Alzheimer’s disease. Multiplexed DNA-labeled Ab imaging by digital spatial profiling (233) of olfactory bulb and olfactory tract tissue from familial Alzheimer’s patients identified viral infection in only the olfactory bulb, yet signatures of inflammation and altered myelination were readily apparent in the olfactory tract (234). This finding may help explain some of the symptoms of Alzheimer’s disease, as the olfactory tract carries information from the olfactory bulb to the hippocampus, which is responsible for memory and learning.

Like SARS-CoV-2, IAV similarly establishes a hyperinflammatory microenvironment, marked by severe innate inflammatory tissue damage that contributes to lethal outcomes (235, 236). Yet, treatment with immunomodulatory drugs has relatively modest effects (235, 237). Reasoning that the limited success of these drugs may be due to a rapid inflammatory response that has passed a point of no return, a multi-omic study found that pairing immunomodulatory drugs that enhance epithelial repair with an antiviral treatment that dampens IAV spread was able to prevent death of infected mice (237). Multiplexed IF imaging by a method termed iterative bleaching extends multiplicity (238, 239) showed that lungs from infected mice treated with anti-IFNAR Ab (IFN response inhibitor) and Tamiflu (antiviral), but not either treatment alone, had increased AT2 cell Ki67 expression and phosphorylated STAT3 (237). These findings are consistent with enhanced alveolar epithelial cell regeneration and tissue repair (240) and highlight the importance of timing for cooperation between immune responses and repair processes.

Multiplexed imaging has also demonstrated the potential for oncolytic viruses to enhance the recruitment of immune cells to an immunosuppressive tumor microenvironment. Multiplexed IF imaging of immune markers showed that intratumoral infiltration of CD8+ T cells increased following treatment with oncolytic strains of HSV-1 and vaccinia virus in CNS and colon tumors, respectively (241, 242). Pairing oncolytic treatment with immune checkpoint inhibitor therapy further enhanced this effect in colon tumors (241) and the same is expected to be true for CNS tumors, as multiplexed IF imaging also revealed an upregulation of checkpoint proteins in this context (242).

Multiplexed imaging has also been applied to identify immune subsets recruited at sites of liver injury in hepatitis B and C patients, finding correlations between the extent of fibrosis and presence of specific immune subsets (243, 244).

### Imaging Mass Spectrometry

In contrast to other imaging approaches that require labeling of analytes for detection, imaging MS (IMS) is an open view tool, allowing simultaneous identification of thousands of different molecules. As such, IMS is not restricted to predefined analytes nor the availability of antibodies to target them. Spatial mapping is achieved by scanning across a tissue section, collecting mass spectra at points within a defined grid, called pixels. The spatial resolution of the image is defined by the pixel size, which varies by IMS method but can reach as low as 600 nm using specialized ionization strategies (245). An array of IMS methods exist (reviewed previously (246)), with the most widely adopted method being matrix-assisted laser desorption/ionization (MALDI)-IMS. In a typical MALDI-IMS experiment, a tissue section of interest is mounted to a specialized slide and coated with a chemical matrix, which absorbs laser energy and promotes analyte desorption and ionization. The sample is irradiated at each pixel with a pulsed laser and a mass spectrum acquired (Fig. 4*C*). MALDI-IMS can measure diverse analytes, such as proteins, metabolites, lipids, and peptides, including proteolytic peptides if proteolytic digestion is performed prior to matrix coating. Microscopy data can also be integrated to guide positioning of pixels to measure single cells (247) and even subcellular features (245, 248). Given its precise spatial resolution and that analyte detection is largely unbiased, IMS represents an excellent discovery tool to probe alterations to tissue caused by viral infection.

IMS has provided mechanistic insights into the causes and effects of inflammation induced during viral myocarditis. Metabolomic profiling by IMS of cardiac biopsies from viral myocarditis patients and a mouse model revealed extensive rewiring of cardiac metabolism, marked by reductions in high-energy phosphates and NAD levels (249). This was accompanied by decreases in protein abundances for several enzymes and other regulators of cardiac oxidative metabolism. Transcriptomics and functional assays revealed that cardiac metabolic remodeling is driven by NF-κB signaling that is activated as a response to cytokines secreted from infiltrating leukocytes (249). In line with this, proteomic profiling by IMS of cardiac biopsies from viral myocarditis patients found a positive association with disease severity and levels of tenascin-C (250), a matricellular protein known to promote a pro-inflammatory cardiac microenvironment and macrophage migration (251). Treatment with the antiviral telbivudine lowered tenascin-C levels and reduced immune infiltration (250). Together, these findings highlight the potential of IMS for uncovering intercellular communication networks that are dysregulated in disease.

IMS has also helped clarify the role of phospholipid biogenesis in HBV-mediated liver disease (252). Through its viral X protein, HBV is known to impair liver regeneration and cause liver disease (253, 254, 255, 256, 257). IMS of liver tissue from an HBV mouse model showed altered composition of several phosphatidylcholines, as well as delayed expression of key phosphatidylcholine biosynthetic enzymes. These changes were associated with liver disease and regeneration defects in the mouse model (252), providing further evidence for the role of phospholipid metabolism in liver regeneration and clarifying mechanisms of HBV-mediated liver disease.

In addition to its uses probing alterations in tissues during infection, IMS also serves as a powerful diagnostic tool for many cancers, including those caused by viruses (258, 259, 260, 261), discriminating with high precision tumor from surrounding healthy tissue.

### Cellular Microenvironment Labeling

Cells within a tissue are highly dynamic. Their spatial positioning relative to each other and interactions with other cells are constantly changing and, in sum, guide cellular states. Given this, it is informative to record a cell’s dynamic microenvironment, tracking which cells have interacted with each other and/or their spatial proximity relationships over time to understand how these interactions influence cellular states and disease outcomes. Such information tends to be missed in tissue sectioning or imaging experiments, as these approaches represent only a snapshot in time. In other instances, the kinds of analyses or depth of omic profiling that is desired cannot be achieved *in situ* in tissue slices, necessitating an ability to isolate cell subpopulations of interest from a microenvironment. Accordingly, an array of technologies has been developed to label cells within a microenvironment. These strategies are based on fluorescent recording of a cell’s interactions with or spatial proximity to other cells (20, 262, 263, 264, 265, 266, 267, 268, 269) (*e.g.*, tumor cell-CD4+ T cell interactions (269) or proximity to infected cells (20)). This enables the isolation of labeled cell subpopulations by fluorescence-assisted cell sorting, followed by diverse downstream analyses, including interrogations that can be performed only *ex situ* such as deep unbiased proteomics or live cell functional assays.

While application of microenvironment labeling strategies has largely centered around the tumor microenvironment and transcriptomic readouts, we recently adapted a spatial proximity labeling technique (262, 268), pairing it with MS-based proteomics to delineate the spatial and functional relationships of cells within a VME (20). Our study demonstrated the value of cellular microenvironment labeling approaches for elucidating unexplored impacts of intercellular communication during a viral infection. The spatial proximity labeling strategy we employed relies on a secreted and cell penetrable version of the mCherry red fluorescent protein (sCP-mCh). By engineering cells to express sCP-mCh and infecting them with a GFP-tagged HCMV, we generated infected microenvironment reporter cells. Co-culturing these infected reporter cells with uninfected WT cells led to spatially resolved labeling of the microenvironment of an HCMV infected cell. Three distinct cell subpopulations were distinguished and isolated: infected cells (GFP+, mCh+), neighbor cells (GFP-, mCh+), and farther away distal cells (GFP-, mCh-) (Fig. 4*D*). Given that the intracellular half-life of sCP-mCh in a neighbor cell is ∼40 h (268), a cell that was adjacent to an infected cell at one point but then moved away remains labeled for some time, providing an element of spatiotemporal memory to the proximity labeling.

By applying this labeling strategy to studying the HCMV microenvironment, we demonstrated that uninfected cells that neighbor an infection site exhibit increased susceptibility to secondary infection by HCMV as well as co-infection by other viruses (20). Several proteome alterations were found to underlie this phenotype in neighbor cells, including dysregulated cell cycle and dampened antiviral immune responses compared to distal cells. Extracellular vesicles were shown to be partly involved in the delivery of select viral proteins to uninfected neighbor cells, providing a possible mechanism for the modulation of cell cycle and immune suppression. In contrast, distal cells had heightened immune responses, being poised to slow the spread of infection. Cell cycle profiling by flow cytometry and infection assays confirmed proteomic data, validating mitotic accumulation of neighbor cells. These findings demonstrate that HCMV can condition a proviral microenvironment that is conducive for viral spread at short range and supportive of co-infections with other viruses, having implications in understanding the oncomodulatory properties of HCMV and infection-linked pathologies.

## Closing Remarks

Across the diverse viruses discussed, intercellular communication as an essential driver of infection outcomes is a common theme. The unique ways in which a virus alters signaling networks between infected and surrounding cells in its VME dictates the extent of viral spread and the severity of disease caused by that infection. Proteomic techniques have been at the heart of characterizing these virus-driven changes. They have provided much needed detail into the precise molecular alterations made to many of the signaling modalities used by infected cells to interface with their local environment. They have additionally shed light into the ways in which these surrounding cells, as spatially organized entities, respond to changes in signaling. By combining the knowledge gained from each of these angles of interrogation, we have achieved a more complete picture of how a virus influences its microenvironment and how these changes, in combination, promote virus spread and/or pathogenesis. This information is critical for the goal of developing therapeutics that minimize the impact of infectious diseases on human health.

Considering the rapid pace of advancement in the proteomics field, emerging technologies are well poised to provide novel insights into intercellular signaling in a VME. For example, a recent LCM-based approach with MS, termed deep visual proteomics, enables spatially resolved and unbiased proteomics of single cells from a tissue slice (209, 270, 271). Application of the deep visual proteomics platform to a VME would invariably uncover interesting biology. Additionally, mapping the architecture of transcellular PPIs formed between two cells (*e.g.*, an immune synapse formed between an infected cell and a T cell) using recent protein nano-environment mapping strategies (140, 141, 142, 143, 144, 145, 146) would likely offer exciting insight into how infected cells dodge immune recognition.

## Conflicts of Interest

The authors declare no competing interests.
